# Cross-Linking of a CD4-Mimetic Miniprotein with HIV-1 Env gp140 Alters Kinetics and Specificities of Antibody Responses against HIV-1 Env in Macaques

**DOI:** 10.1128/JVI.00401-17

**Published:** 2017-09-12

**Authors:** Xiaoying Shen, Willy M. Bogers, Nicole L. Yates, Guido Ferrari, Antu K. Dey, William T. Williams, Frederick H. Jaeger, Kevin Wiehe, Sheetal Sawant, S. Munir Alam, Celia C. LaBranche, David C. Montefiori, Loic Martin, Indresh Srivastava, Jonathan Heeney, Susan W. Barnett, Georgia D. Tomaras

**Affiliations:** a Duke Human Vaccine Institute, Duke University Medical Center, Durham, North Carolina, USA; b Department of Medicine, Duke University Medical Center, Durham, North Carolina, USA; c Department of Immunology, Duke University Medical Center, Durham, North Carolina, USA; d Department of Molecular Genetics and Microbiology, Duke University Medical Center, Durham, North Carolina, USA; e Department of Surgery, Duke University Medical Center, Durham, North Carolina, USA; f Biomedical Primate Research Centre, Rijswijk, The Netherlands; g Commissariat à l'Energie Atomique, iBiTecS, Service d'Ingénierie Moléculaire des Protéines, Gif-sur-Yvette, France; h Protein Sciences Corporation, Meriden, Connecticut, USA; i Lab of Viral Zoonotics, Cambridge University, Cambridge, England; j Novartis Vaccines and Diagnostics, Inc., Cambridge, Massachusetts, USA; Ulm University Medical Center

**Keywords:** CD4 mimetic, antibody, epitope exposure, human immunodeficiency virus, nonhuman primate, structural modification, vaccine

## Abstract

Evaluation of the epitope specificities, locations (systemic or mucosal), and effector functions of antibodies elicited by novel HIV-1 immunogens engineered to improve exposure of specific epitopes is critical for HIV-1 vaccine development. Utilizing an array of humoral assays, we evaluated the magnitudes, epitope specificities, avidities, and functions of systemic and mucosal immune responses elicited by a vaccine regimen containing Env cross-linked to a CD4-mimetic miniprotein (gp140-M64U1) in rhesus macaques. Cross-linking of gp140 Env to M64U1 resulted in earlier increases of both the magnitude and avidity of the IgG binding response than those with Env protein alone. Notably, IgG binding responses at an early time point correlated with antibody-dependent cellular cytotoxicity (ADCC) function at the peak immunity time point, which was higher for the cross-linked Env group than for the Env group. In addition, the cross-linked Env group developed higher IgG responses against a linear epitope in the gp120 C1 region of the HIV-1 envelope glycoprotein. These data demonstrate that structural modification of the HIV-1 envelope immunogen by cross-linking of gp140 with the CD4-mimetic M64U1 elicited an earlier increase of binding antibody responses and altered the specificity of the IgG responses, correlating with the rise of subsequent antibody-mediated antiviral functions.

**IMPORTANCE** The development of an efficacious HIV-1 vaccine remains a global priority to prevent new cases of HIV-1 infection. Of the six HIV-1 efficacy trials to date, only one has demonstrated partial efficacy, and immune correlate analysis of that trial revealed a role for binding antibodies and antibody Fc-mediated effector functions. New HIV-1 envelope immunogens are being engineered to selectively expose the most vulnerable and conserved sites on the HIV-1 envelope, with the goal of eliciting antiviral antibodies. Evaluation of the humoral responses elicited by these novel immunogen designs in nonhuman primates is critical for understanding how to improve upon immunogen design to inform further testing in human clinical trials. Our results demonstrate that structural modifications of Env that aim to mimic the CD4-bound conformation can result in earlier antibody elicitation, altered epitope specificity, and increased antiviral function postimmunization.

## INTRODUCTION

A critical component in the path toward the development of a successful human immunodeficiency virus type 1 (HIV-1) vaccine strategy is the definition of the epitope specificities, locations (systemic or mucosal), and effector functions of antibodies elicited by novel HIV-1 immunogens engineered to improve exposure of specific epitopes. There is a growing body of evidence from animal models showing that antibodies can control virus replication (1–4) through elimination of infected cells (4), engagement of Fc-mediated antibody effector functions to limit founder viruses (2), and delay of acquisition and/or prevention of the establishment of infection (5–15) through mechanisms including virus neutralization (8–14, 16) and antibody Fc-mediated antiviral functions (11, 15, 17). Together, these studies include both passive immunization strategies and vaccine approaches that have tested a range of antibody specificities, antibody isotypes, and effector functions (broadly neutralizing, non-broadly neutralizing, and antibody Fc-mediated antiviral activities), thus demonstrating that there is much diversity in the types of antibodies that may protect. However, there remains a gap in understanding how different immunogen designs specifically affect antibody specificities, kinetics, and antiviral functions (i.e., neutralizing and non-broadly neutralizing activities).

There are numerous challenges for inducing broadly neutralizing antibody functions by vaccination, including but not limited to shielding of key epitopes by glycans, difficulty in presentation of the correct Env structures, and the unusual traits of broadly neutralizing antibodies (18, 19). In contrast, the one HIV-1 vaccine that was partially efficacious in humans demonstrated a potential role for non-broadly neutralizing antibodies in preventing HIV-1 acquisition (20). Non-broadly neutralizing antibodies include CD4-induced (CD4i) antibodies that target epitopes whose exposure is triggered by binding of HIV-1 Env gp120 to CD4 on the host cell. A recent study demonstrated that CD4i antibodies were correlated with viremia control following mucosal challenge in rhesus macaques (3).

HIV vaccine strategies can involve modifying the structure of Env for improved exposure of CD4i epitopes. CD4i epitopes include coreceptor binding sites (21, 22) that are highly conserved (23–25) and variable loop domains (26, 27), some of which are easily elicited during natural HIV-1 infection (24, 28, 29). One immunogen design approach utilizes coexpression of CD4 in a single molecular structure with HIV-1 Env to promote binding and complex formation of CD4 and Env (3, 30–34). Another approach involves small-molecule CD4-mimetic compounds, which have been shown to inhibit HIV-1 entry by competitively binding to the CD4 binding site (CD4bs) (35, 36). A recent study further showed that CD4-mimetic compounds can activate or inactivate primary HIV Env trimers, depending on the properties of the CD4 mimetics and the Env trimer and how many subunits of the trimer are bound (37). Several studies have explored biochemical cross-linking of synthetic CD4-mimetic molecules with Env proteins for improved CD4i epitope exposure (31, 38–41). In particular, the CD4-mimetic miniprotein M64U1 has been shown to expose both CD4i epitopes and coreceptor binding sites when covalently conjugated to Env gp140 (38), eliciting increased titers of CD4i antibody-mediated neutralization in rabbit immunization studies (38, 42). The gp140-M64U1 cross-linked vaccine was further tested in macaques (69) and was shown to alter the kinetics of B cell responses and the levels of neutralization and antibody-dependent cellular cytotoxicity (ADCC) responses. In the present study, we further characterized the magnitudes, specificities, and kinetics of binding antibody responses and examined the correlation between these parameters of binding antibody responses and antibody functions, providing novel evidence that the cross-linked gp140-M64U1 complex can affect both the binding properties of and the antiviral functions mediated by Env-specific antibodies in primates.

## RESULTS

Rhesus macaques were immunized with a gp140 protein with variable loop 2 (V2) deleted (SF162 gp140ΔV2) five times, either alone (gp140 group; 6 animals) or cross-linked with a CD4-mimetic miniprotein (gp140-M64U1 group; 6 animals) (69) (Table 1). Env-specific antibody responses, including systemic and mucosal binding specificities and antibody avidities, were evaluated with samples collected at week 6 (2 weeks after the 2nd immunization), week 26 (2 weeks after the 3rd immunization), week 38 (2 weeks after the 4th immunization), and week 107 (the time of the 5th immunization, that is, 71 weeks after the 4th immunization).

**TABLE 1:** Immunization groups^*a*^

Group	Immunogen	Dose (mg)
1	SF162 gp140ΔV2	100
2	M64U1	50
3	M64U1-SF162 gp140ΔV2	100
4	None	

a All groups (*n* = 6 animals/group) received MF59 as an adjuvant, and all vaccine doses were administered intramuscularly at 0, 4, 24, 36, and 107 weeks.

### Early Env-binding IgG response with gp140-M64U1 vaccine.

**FIG 1:**
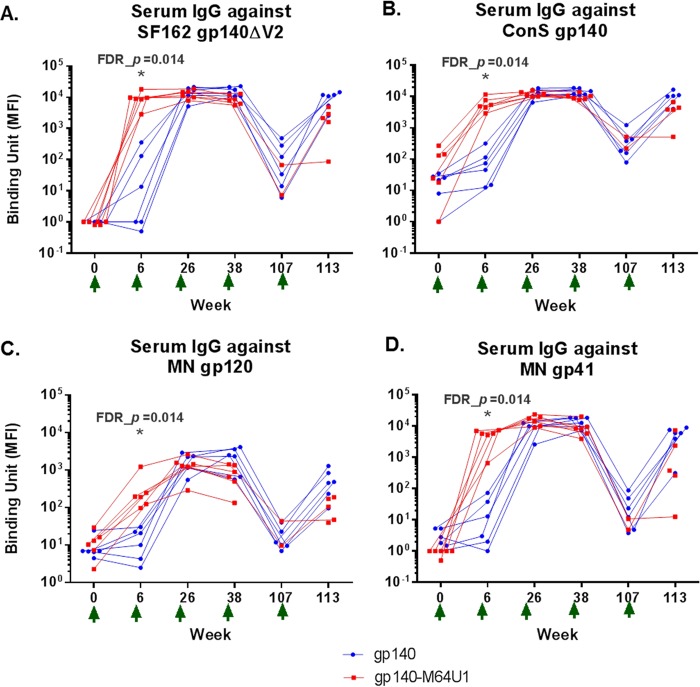
Longitudinal antibody binding responses for SF162 gp140ΔV2 (vaccine strain) (A), ConS gp140 (B), MN gp120 (C), and MN gp41 (D). The data shown are MFI binding values within the linear range of the assay for each antigen (1:400 for SF162 gp140ΔV2 and MN gp41 and 1:80 for ConS gp140 and MN gp120). The gp140 group data are shown in blue, and the gp140-M64U1 group data are shown in red. For improved data visualization of data points with similar magnitudes, the *x* axis was plotted categorically with staggered symbols so that each data point is visible. One animal in the gp140-M64U1 group died before week 107, and serum samples were not available for another 3 animals in the gp140-M64U1 group at week 107, therefore leaving 2 data points for the gp140-M64U1 group for week 107 and 5 data points for this group for week 113. Green arrows below the *x* axis indicate times of vaccination. FDR_*p*, Wilcoxon rank sum exact test *P* value controlled for FDR by the Benjamini-Hochberg method. *, FDR_*P* < 0.05.

To characterize the development of Env-specific binding antibody responses over time, we tested longitudinal serum samples from the vaccinated animals for binding to the SF162 gp140ΔV2 (the immunogen), ConS gp140 (group M consensus [43–45]), MN gp120, and MN gp41 proteins in binding antibody multiplex assays (BAMA). Among the 4 Env antigens tested, the highest response was seen for binding to SF162 gp140ΔV2 (the vaccine strain), followed by ConS gp140. Similar kinetics were observed for the development of the Env-specific IgG responses against the 4 Env antigens examined. Serum IgGs specific for the Env proteins were detectable as early as week 6 (2 weeks after the 2nd immunization) for all 4 Env antigens tested for both the gp140 and gp140-M64U1 groups (Fig. 1A to D). The responses generally peaked at week 26 (2 weeks after the 3rd immunization), with week 38 (2 weeks after the 4th immunization) levels being comparable to those for week 26 for both groups. The responses measured at week 107 (71 weeks after the 4th immunization) declined, as expected, followed by a boost in the responses measured at week 113 (6 weeks after the 5th immunization) (Fig. 1A to D).

While the peak levels of binding antibody responses (weeks 26 and 38) were generally comparable between the two groups, the gp140-M64U1 group showed significantly higher binding antibody responses at week 6, revealing faster kinetics in the development of the anti-Env responses. For all 4 Env proteins tested, binding by the week 6 sera was much higher for the gp140-M64U1 group than for the gp140 group, with an FDR_*P* value (Wilcoxon rank sum exact test *P* value controlled for the false discovery rate [FDR] by the Benjamini-Hochberg method) of 0.014 (Fig. 1A to D; Table 2). Binding responses to all 4 Env proteins were again comparable between the gp140 and gp140-M64U1 groups after the last immunization, at week 113 (Fig. 1A; Table 2).

**TABLE 2:** Between-group comparisons with FDR-controlled *P* values^*a*^

Group comparison and protein/time point	Raw *P* value	FDR_*P* value
Serum IgG binding BAMA (MFI), gp140 group vs gp140-M64U1 group		
SF162 gp140ΔV2/wk 6	**0.002**	0.014*
SF162 gp140ΔV2/wk 26	0.485	0.614
SF162 gp140ΔV2/wk 38	**0.026**	0.071
SF162 gp140ΔV2/wk 113	**0.017**	0.052
ConS gp140/wk 6	**0.002**	0.014*
ConS gp140/wk 26	0.818	0.897
ConS gp140/wk 38	**0.015**	0.052
ConS gp140/wk 113	**0.017**	0.052
MN gp120/wk 6	**0.002**	0.014*
MN gp120/wk 26	0.818	0.897
MN gp120/wk 38	0.093	0.189
MN gp120/wk 113	**0.030**	0.075
MN gp41/wk 6	**0.002**	0.014*
MN gp41/wk 26	0.180	0.277
MN gp41/wk 38	0.180	0.277
MN gp41/wk 113	0.126	0.231
C1_104.AE/wk 6	0.180	0.277
C1_104.AE/wk 26	**0.002**	0.014*
C1_104.AE/wk 38	**0.015**	0.052
C1_104.AE/wk 113	0.082	0.180
Serum IgG avidity SPR (off-rate [*K_d_*]), gp140 group vs gp140-M64U1 group		
SF162 gp140ΔV2/wk 6	**0.004**	0.022*
SF162 gp140ΔV2/wk 26	0.699	0.813
SF162 gp140ΔV2/wk 38	**0.041**	0.098
SF162 gp140ΔV2/wk 113	**0.017**	0.052
Serum IgG ADCC^*b*^ (titer), gp140 group vs gp140-M64U1 group		
SF162 gp140ΔV2/wk 26	**0.002**	0.014*
SF162 gp140ΔV2/wk 113	0.126	0.231
Serum neutralization^*b*^ (ID_50_), gp140 group vs gp140-M64U1 group		
SHIV-SF162P4/wk 38	**0.026**	0.071
SHIV-SF162P4/wk 42	0.506	0.628
SHIV-SF162P4/wk 113	0.126	0.231
Serum linear epitope mapping (signal intensity), gp140 group vs gp140-M64U1 group		
C1.1/wk 26	**0.028**	0.073
C1.2/wk 26	**0.009**	0.038*
C2/wk 26	0.318	0.422
V3/wk 26	0.937	0.948
C4/wk 26	0.387	0.502
V5-C5/wk 26	0.242	0.343
C5.1/wk 26	0.180	0.277
C5.2/wk 26	0.937	0.948
gp41-ID/wk 26	0.240	0.343
gp160 total/wk 26	0.093	0.189
CD4bs panel BAMA (WT/mutant ratio), gp140 group vs gp140-M64U1 group		
RSC3 WT:Δ371/wk 26	0.536	0.650
YU gp120 core WT:D368R/wk 26	**0.043**	0.099
Serum IgA binding BAMA (MFI), gp140 group vs gp140-M64U1 group		
SF162 gp140ΔV2/wk 6	0.180	0.277
SF162 gp140ΔV2/wk 26	0.937	0.948
SF162 gp140ΔV2/wk 38	0.310	0.420
SF162 gp140ΔV2/wk 113	0.247	0.343
Nasal IgG binding BAMA (sp act), gp140 group vs gp140-M64U1 group		
SF162 gp140ΔV2/wk 38	0.132	0.235
Nasal IgG binding BAMA (sp act), gp140 group vs mock control group		
SF162 gp140ΔV2/wk 38	**0.004**	0.022*
Nasal IgG binding BAMA (sp act), gp140-M64U1 group vs M64U1 control group		
SF162 gp140ΔV2/wk 38	**0.015**	0.052

a Raw *P* value, Wilcoxon rank sum exact test *P* value, not corrected for multiple comparisons; FDR_*P* value, Wilcoxon rank sum exact test *P* value controlled for FDR, calculated according to the Benjamini-Hochberg method (68). FDR was performed across Wilcoxon rank sum tests for Table 2 and across Spearman correlation tests for Table 3 (57 tests in total). Values in bold are *P* values of <0.05. *, significant difference between groups (FDR_*P* < 0.05).

b Quantification of neutralization and ADCC responses is reported by Bogers et al. (69).

### Decreased linear C1 epitope IgG with gp140-M64U1 vaccine.

**FIG 2:**
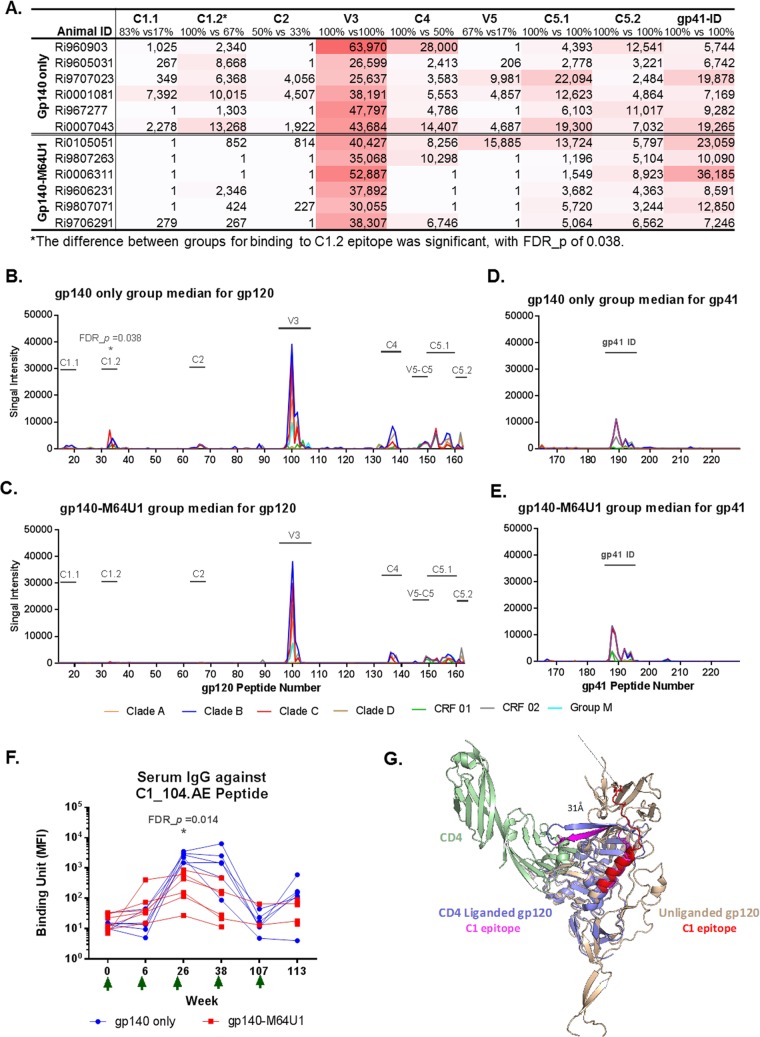
Linear epitope specificity of serum IgG by epitope mapping (A to E) and BAMA (F). Week 26 mean binding intensity values for serum IgG for the gp140-only (B and D) and gp140-M64U1 (C and E) groups are shown for overlapping peptides of 7 consensus gp120 (B and C) and gp41 (D and E) sequences. Different colors represent different clades/circular recombinant forms (CRFs). Epitope regions identified in the study are indicated by text over a horizontal bar in plots. (A) Magnitude of binding to each epitope, calculated as the highest level of binding to a single peptide within each epitope region. The percentages listed for each epitope are the response rates to the epitope by the animals of the 2 groups (gp140 versus gp140-M64U1). The peptide ranges for the epitopes are as follows: C1.1, residues 16 to 21; C1.2, residues 32 to 39; C2, residues 65 to 68; V3, residues 97 to 104; C4, residues 133 to 139; V5-C5, residues 147 to 151; C5.1, residues 152 to 159; C5.2, residues 161 to 163; and gp41-ID, residues 187 to 194. The sequences of all peptides have been published previously (65). (F) Longitudinal binding to the C1_104.AE peptide (corresponds to the C1.2 epitope in epitope mapping) was measured by BAMA. Green arrows indicate times of immunization. (G) Structural modeling of the conformational change of the C1 epitope upon CD4 binding. The C1_104 epitope bends ∼90° from the unliganded gp120 conformation (gp120 monomer from SOSIP Env trimer; PDB entry 4TVP) (beige) to the CD4-liganded gp120 conformation (PDB entry 4RQS) (light blue). Binding of CD4 (PDB entry 4QRS; green) results in a >30-Å displacement of the C-terminal residue (stick representation) between the C1_104 epitope in the unliganded gp120 protein (red) and the CD4-bound C1_104 epitope (magenta).

Week 26 (the peak immunity time point, 2 weeks after the 2nd immunization) serum samples from all immunized animals were profiled for binding antibodies against gp160 linear epitopes by use of a peptide microarray. The HIV-1 Env peptide library contained overlapping peptides covering 7 full-length consensus gp160 sequences (clades A, B, C, and D, group M, CRF01, and CRF02). Serum IgGs from both the gp140 and gp140-M64U1 groups bound epitopes in the C1, C2, V3, C4, V5, and C5 regions of gp120 (Fig. 2A to C) and the immunodominant (ID) region of gp41 (Fig. 2A, D, and E). The magnitudes of binding to these epitopes were generally comparable between the 2 immunized groups at week 26 (Fig. 2A), with the exception of epitope C1.2 binding, which was significantly higher for the gp140 group than for the gp140-M64U1 group (FDR_*P* = 0.038) (Fig. 2B and C; Table 2). Interestingly, the C1.2 linear epitope was identified in epitope mapping studies of the RV144 Thai trial, and plasma IgA binding to the corresponding C1 peptide covering the entire epitope region (C1_104.AE [MQEDVISLWDQSLKPCVKLTPLCV]) correlated with an increased risk of HIV-1 infection (i.e., decreased vaccine efficacy) in the secondary/exploratory immune correlate analysis of the trial (20). To further evaluate the kinetics and magnitude of this response, we measured the serum IgG response to the linear C1_104.AE peptide over time by BAMA. The binding response against C1_104.AE was significantly higher for the gp140 group than for the gp140-M64U1 group at week 26 (FDR_*P* = 0.014) (Fig. 2F; Table 2), consistent with the trend observed in the week 26 linear epitope mapping data (Fig. 2A to C). Similar to the binding responses against Env proteins, binding responses to C1_104.AE peaked at weeks 26 and 38, declined at week 107, and then increased again at week 113, after the fifth immunization.

We modeled the C1_104 epitope in the monomeric subunit of gp120 from the SOSIP Env trimer structure (46), which is representative of the prefusion conformation, and found that it is exposed on the monomeric gp120 surface (Fig. 2G, red ribbon). In the CD4-bound state, the formation of the bridging sheet results in the C-terminal half of the C1_104 epitope bending ∼90° relative to the epitope in the SOSIP structure (Fig. 2G, pink ribbon). This bending results in a >30-Å displacement in the position of the C-terminal residue of the C1_104 epitope, which in turn contacts CD4 (47). Given the large conformational change and associated burial of C-terminal residues upon contacting CD4, antibodies that recognize C1_104 in the unbound conformation may therefore not be able to recognize the epitope in the CD4-bound state. While the effect of CD4 binding on the conformation of the C1_104 epitope in a V2-deleted gp140 protein may be different, these structural data do suggest that the CD4bs-cross-linked antigen may substantially affect the exposure of the C1 epitope on the Env immunogen.

### Induction of CD4bs antibodies by vaccination.

**FIG 3:**
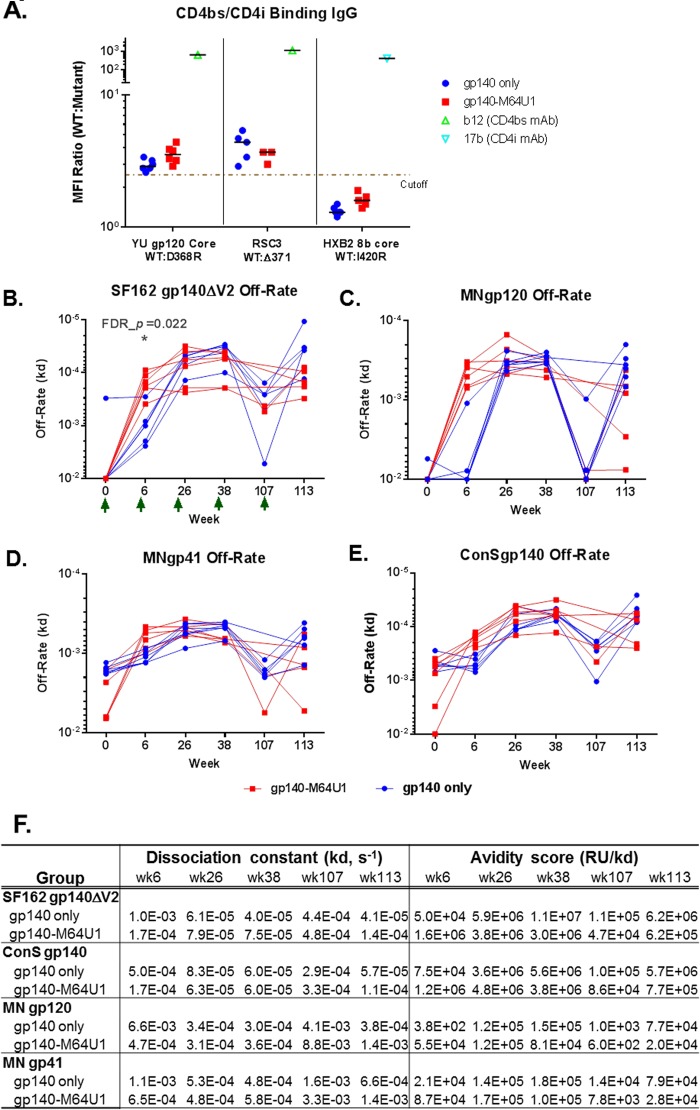
CD4bs and CD4i specificities (A) and off-rate measurements for SF162 gp140ΔV2 (B), MN gp120, (C), MN gp41 (D), and ConS gp140 (E) for the 2 vaccine groups, with group mean off-rates and avidity score values as measured by SPR (F). The cutoff for the CD4bs and CD4i differential binding assay was 2.5-fold. For the CD4bs/CD4i differential binding assay, b12 (CD4bs monoclonal antibody [MAb]) was used as a positive control for YU gp120 core WT/D368R differential binding and RSC3 WT/Δ371 differential binding (48), and 17b (CD4i MAb) was used as a positive control for HXB2 8b core WT/I420R differential binding (28, 48). Serum samples were tested at 1:400. Control MAbs b12 and 17b were tested at 25 and 50 μg/ml, respectively. All baseline serum samples were negative for binding to both the WT and mutant proteins in this test panel. Green arrows indicate times of immunization. FDR_*p*, Wilcoxon rank sum exact test *P* value controlled for FDR by the Benjamini-Hochberg method. *, FDR_*P* < 0.05. Between-group comparison test results are shown in Table 2.

Since the design of the gp140-M64U1 cross-linked immunogen involved potential modifications of gp120-CD4 interactions, we evaluated the levels of CD4 binding site (CD4bs) and CD4-inducible (CD4i) antibodies by measuring binding of the antibodies to gp120 structures with and without mutations that are known to interfere with recognition of CD4bs and CD4i epitopes (48). In particular, the D368R mutation abrogates binding of most CD4bs antibodies to gp120 core or gp120 (28, 48–51), the Δ371 mutation abrogates binding of VRC01-like antibodies to the gp120 resurfaced stabilized core protein (RSC3) (48, 50), and the I420R mutation abrogates binding of gp120 core to 17b-like CD4i antibodies (28, 50). With these reagents, we detected CD4bs-binding antibodies (indicated by YU gp120 core wild type [WT]/D368R ratios of ≥2.5) in both vaccination groups (Fig. 3A), with comparable levels between the 2 groups (Table 2). Furthermore, VRC01-like binding antibodies (indicated by RSC3 WT/Δ371 ratios of ≥2.5) developed in both vaccination groups, at generally comparable levels (Fig. 3A; Table 2). 17b-like CD4i antibodies, defined by HXB2 8b core WT/I420R ratios of ≥2.5, were not induced (Fig. 3A).

### Early increase in antibody avidity with gp140-M64U1 vaccine.

Maturation of the vaccine-elicited antibody response through determination of HIV-1 Env antibody avidity is an indicator of the quality of the vaccine-induced antibody responses. We measured the avidities (using the dissociation rate constant [off-rate] [*K_d_*]) and avidity scores (response units [RU]/*K_d_*) of purified serum IgGs (from weeks 6, 26, 38, 107, and 113) for binding to SF162 gp140ΔV2 (Fig. 3B), MN gp120 (Fig. 3C), MN gp41 (Fig. 3D), and ConS gp140 (Fig. 3E). The avidity scores of serum IgGs to these antigens peaked at weeks 26 and 38 (Fig. 3F), and off-rates dropped to their lowest levels at the same time points (Fig. 3B to F). At week 6, the off-rate for SF162 gp140ΔV2 was significantly lower for the gp140-M64U1 group than for the gp140 group (medians of 1.7 × 10^−4^ and 1.0 × 10^−3^ s^−1^ for the gp140-M64U1 and gp140 groups, respectively; FDR_*P* = 0.022) (Fig. 3B; Table 2). Off-rates were not statistically different, after FDR correction, for the two vaccine groups at weeks 26, 38, and 113 (Fig. 3B to F; Table 2). The longitudinal patterns of antibody off-rates and avidity scores for gp41, gp120, and ConS gp140 were similar to those for SF162 gp140ΔV2, with the gp140-M64U1 group trending toward having a lower off-rate (Fig. 3C to E) and a higher avidity score (Fig. 3F) than those of the gp140 group.

### Serum IgG Env binding avidity and magnitude correlate with ADCC and neutralization.

**FIG 4:**
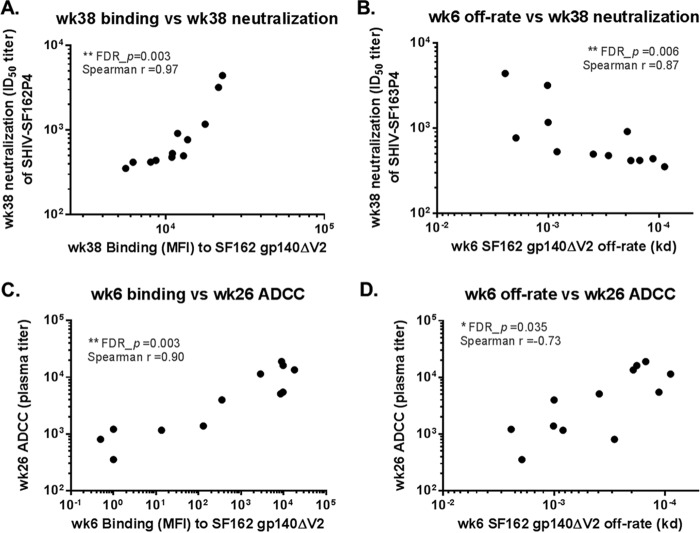
Correlation of neutralization (A and B) and ADCC (C and D) activities with serum IgG binding magnitude (A and C) and serum IgG avidity (B and D). Spearman correlation analysis was performed using SAS, and *P* values were corrected for FDR across all between-group comparison tests (Table 2) and this correlation test (Table 3). **, FDR_*P* < 0.01; *, FDR_*P* < 0.05. ID_50_ titer, 50% inhibitory dose titer.

We further explored correlations between binding antibody properties (binding mean fluorescence intensities [MFI] and off-rates) and antiviral functions (neutralization and ADCC) of the antibodies elicited in the study. Vaccinations elicited low to moderate levels of neutralizing antibodies against SF162P4, with titers ranging from <10 to 4,403 at the peak neutralizing activity time point of week 38 (post-3rd immunization) in most animals (69). Neutralization of simian-human immunodeficiency virus (SHIV) SF162P4 at week 38 was found to correlate significantly with week 38 serum IgG binding to SF162 gp140ΔV2 (FDR_*P* = 0.003; Spearman *r* = 0.97) (Fig. 4A; Table 3). Week 38 neutralization of SHIV SF162P4 was also found to correlate with a higher off-rate for SF162 gp140ΔV2 at week 6 (FDR_*P* = 0.006; Spearman *r* = 0.87) (Fig. 4B), which indicated an inverse correlation with avidity; however, week 38 neutralization was not significantly correlated with the contemporaneous (week 38) off-rate for SF162 gp140ΔV2 (Table 3).

**TABLE 3:** Spearman correlation test with FDR control for correlations between antibody functions (ADCC or neutralization) and binding antibody responses (avidity or IgG binding) and between nasal and serum IgG responses^*a*^

Parameter 1	Parameter 2	Raw *P* value	FDR_*P* value	Spearman *r* value
wk 26 ADCC (linear titer)	wk 6 off-rate (*K_d_*)	**0.0074**	0.035*	−0.73
	wk 26 off-rate (*K_d_*)	0.95	0.948	−0.021
	wk 6 IgG binding (BAMA MFI)	**<0.0001**	0.003*	0.9
	wk 26 IgG binding (BAMA MFI)	0.91	0.948	0.035
wk 38 neutralization (ID_50_ titer)	wk 6 off-rate (*K_d_*)	**0.0003**	0.006*	0.87
	wk 38 off-rate (*K_d_*)	0.75	0.850	−0.1
	wk 6 IgG binding (BAMA MFI)	0.2	0.298	−0.4
	wk 38 IgG binding (BAMA MFI)	**<0.0001**	0.003*	0.97
Nasal IgG (SA)	Serum IgG binding (MFI)	0.56	0.661	0.19

a Raw *P* value, Spearman rank correlation test *P* value, not corrected for multiple comparisons; FDR_*P* value, Spearman rank correlation test *P* value controlled for the FDR, calculated according to the Benjamini-Hochberg method (68). FDR was performed across Wilcoxon rank sum tests for Table 2 and across Spearman correlation tests for Table 3 (57 tests in total). Values in bold are *P* values of <0.05. *, significant correlation after controlling for FDR (FDR_*P* < 0.05).

Both vaccine groups developed strong ADCC activity as measured with SF162 gp120-coated cells, which peaked at week 26, after the 2nd immunization, with titers of up to 19,024 (69). ADCC titers were significantly higher for the gp140-M64U1 group than for the gp140 group (FDR_*P* = 0.014) at week 26, and they trended higher at week 113 (Table 2). Correlation analysis revealed that ADCC activity at week 26 (post-3rd immunization) did not correlate with either the contemporary (week 26) binding magnitude or avidity for SF162 gp140ΔV2 but rather correlated with week 6 (post-2nd immunization) serum IgG binding (FDR_*P* = 0.003; Spearman *r* = 0.90) (Fig. 4C) and avidity (FDR_*P* = 0.035; Spearman *r* = −0.73 for off-rate) (Fig. 4D; Table 3) for SF162 gp140ΔV2, indicating that binding antibody responses early on may predict later antibody functions following further immunizations.

### Low levels of serum IgA elicited.

**FIG 5:**
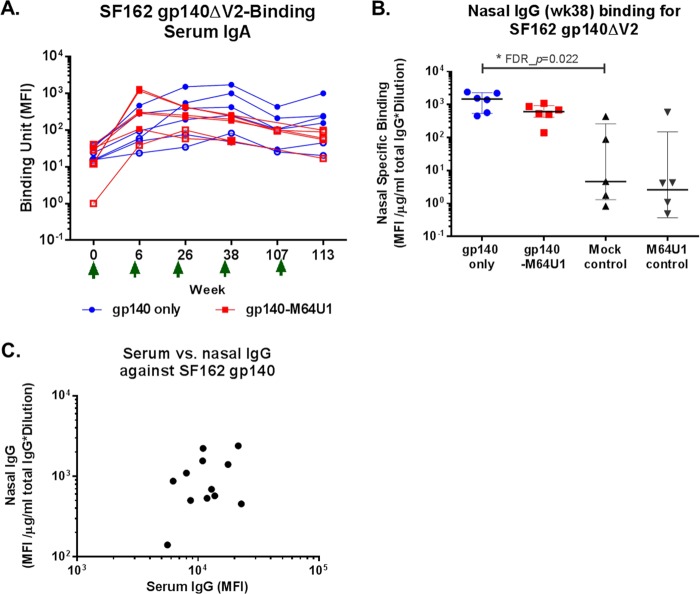
Longitudinal binding of serum IgA to SF162 gp140ΔV2 (A), week 38 nasal IgG binding to SF162 gp140ΔV2 (B), and correlation between levels of serum and nasal IgG binding to SF162 gp140ΔV2 at week 38 (C). MFI binding values shown for IgA binding are for a 1:80 serum dilution. Binding specificities for nasal samples were normalized to the total IgG concentration in each sample. Green arrows indicate times of immunization. *, FDR_*P* < 0.05.

Env-specific IgA responses were evaluated in longitudinal serum samples. The overall magnitudes of HIV-1 Env serum IgA responses were much lower than those of the serum IgG responses (Fig. 5A versus Fig. 1A), with an IgA binding positivity rate of 66.7% at week 26 for SF162 gp140ΔV2 for both the gp140 and gp140-M64U1 groups, compared to 100% IgG binding to SF162 gp140ΔV2 at week 26. Similar to serum IgG responses, serum IgA binding to SF162 gp140ΔV2 peaked earlier for the gp140-M64U1 group, at week 6, than for the gp140 group, with a peak at week 26 (Fig. 5A). However, no significant difference in the magnitude of responses was detected between the two groups for week 6 or any other time point (Fig. 5A; Table 2).

### Vaccine-elicited mucosal antibody responses.

Nasal and rectal samples were collected from vaccinated animals at week 38. Env-specific IgG responses were evaluated in these mucosal samples by use of BAMA (29, 52). The binding magnitude (MFI) was normalized to the total recovered rhesus IgG concentration (in micrograms per milliliter) in each mucosal sample to account for sampling variations. Total rhesus IgG concentrations ranged from <0.5 to 109 μg/ml (median, 9.1 μg/ml) and from <0.5 to 165 μg/ml (median, 9.9 μg/ml) for nasal and rectal samples, respectively. We detected Env-specific IgGs against SF162 gp140ΔV2, MN gp120, and MN gp41 in nasal washes from both the gp140 and gp140-M64U1 groups, with comparable magnitudes (Fig. 5B and data not shown). Compared to samples from mock-immunized control animals, nasal samples from the gp140 group showed significantly higher levels of SF162 gp140ΔV2-specific antibodies (FDR_*P* = 0.022) (Table 2). The specific binding of IgGs from the rectal washes of the vaccinated animals to these Env proteins was not statistically different from that of control animals (data not shown). We further examined the correlation between IgG responses in the serum and mucosal compartments and found a lack of significant correlation between serum and nasal IgG responses for binding to SF162 gp140ΔV2 (Fig. 5C), indicating that these are distinct immune measurements.

## DISCUSSION

Here we report the detailed binding specificities, avidities, kinetics, and functional correlations of antibodies generated by immunization of rhesus macaques with an HIV-1 envelope protein (SF162 gp140ΔV2) cross-linked with a CD4-mimetic miniprotein, M64U1 (69). Our findings demonstrate that cross-linking of the CD4-mimetic M64U1 miniprotein with gp140 significantly affects the kinetics, binding specificity, avidity, and ADCC activity of the vaccine-elicited antibodies compared to those seen with the gp140 protein alone. Comparison of the binding antibody responses between the gp140 and gp140-M64U1 groups revealed an accelerated development of anti-Env binding responses in the gp140-M64U1 group, as indicated by higher levels of binding to the gp120 and gp140 Env proteins than those in the gp140-only group at week 6 (post-2nd immunization) (Fig. 1; Table 2). However, Env binding responses became comparable by the time the antibody responses peaked (at weeks 26 and 38 [post-3rd and -4th immunization, respectively]), whereas binding to a linear C1 epitope was higher for the gp140 group than for the gp140-M64U1 group at week 26. Antibody responses against M64U1 or CD4 were not measured in the current study. Follow-up studies could examine whether anti-immunogen responses were elicited and could have affected gp140 antibody responses in the gp140-M64U1 group following later boosts.

In a previous study with rabbits (42), the gp140-M64U1 complex elicited significant levels of CD4i antibodies as measured by absorption/depletion with gp120 proteins carrying the I420R mutation, which is critical for binding by 17b-like CD4i antibodies, and by neutralization of an HIV-1 strain with and without the presence of soluble CD4. In the current study with nonhuman primates (NHPs), no significant 17b-like CD4i antibody responses were detected in either the gp140 or gp140-M64U1 group during examination of the differential binding of serum to gp120 core proteins with and without the I420R mutation (Fig. 3A). Another difference between the previous rabbit study and the current macaque study is the higher levels of neutralizing antibodies directed to the CD4i epitopes following gp140-M64U1 immunization observed in the rabbit study, but not in the current macaque study (69). Apart from differences in study methods, species differences may play a role in the difference observed. Macaques have intrinsic expression of CD4 molecules along with other surface molecules, including coreceptor and DC-SIGNs (53–55), that may interact with SIV and HIV Env, which likely affects the responses of macaques to a mini-CD4-cross-linked Env. One concern about the use of CD4-mimetic proteins in vaccine regimens is the potential effect on the development of CD4bs antibodies. Broadly neutralizing CD4bs antibodies have been shown to recognize a site of “vulnerability” on HIV-1 Env (56). Binding antibodies directed to CD4bs are commonly induced in HIV infection (48), but unfortunately, those with broadly neutralizing activity seem to develop in a smaller subset of individuals (48, 57). CD4bs antibodies were detected in the gp140 group in the rabbit study (42). In the current study, we also found comparable levels of CD4bs antibodies in both the gp140 and gp140-M64U1 groups (Fig. 3A).

One surprising finding in this study was the impact of M64U1-Env cross-linking on the kinetics, specificity, and avidity of antibody responses. Both the binding magnitude and avidity of the Env-specific antibodies were significantly higher (FDR_*P* = 0.014) for the gp140-M64U1 group at week 6 (post-2nd immunization), although the two groups were comparable or the gp140 group trended higher than the gp140-M64U1 group at later time points (Fig. 1A to D and 3C; Table 2). The mechanisms for the faster development of antibody responses in the gp140-M64U1 group are not clear and warrant further investigation, including whether cross-linking of M64U1 and gp140 can affect the stability and *in vivo* trafficking of the Env protein and how cross-linking with M64U1 affects the interaction of Env with cells of both the adaptive and innate immune systems. In particular, exploring the B cell responses in this macaque study revealed larger proportions of Env-specific B cells in peripheral blood mononuclear cells (PBMC) (69). It was hypothesized that cross-linking with M64U1 interferes with CD4 receptor engagement, thus improving CD4 T cell-dependent immune responses.

In contrast to the binding results with the Env proteins, binding to a C1 epitope, C1_104 (MQEDVISLWDQSLKPCVKLTPLCV; the sequence matches the AE clade consensus sequence), was significantly higher for the gp140 group than for the gp140-M64U1 group at the peak immunity time point of week 26, as shown by both a linear epitope mapping microarray and BAMA (Fig. 2A, B, and F; Table 2). Plasma IgA responses to the same epitope were positively correlated with HIV-1 risk in the human RV144 vaccine clinical trial (20). Quantification of the anti-C1.2 IgA response was not possible in the present study due to the low levels of overall IgA responses. Characterization of IgA responses against this C1 epitope and their correlation with the ADCC response warrant further investigation. In addition, even though conformational C1-binding IgA has been indicated to potentially block IgG-mediated ADCC activity (58) and monoclonal IgG antibodies targeting conformational C1 epitopes can synergize with V2 antibodies to increase ADCC and neutralizing activities (59), the role of C1 linear epitope-binding IgG in vaccine protection is not yet understood.

Another interesting finding of this study was the significantly higher ADCC activity in the sera of animals in the gp140-M64U1 group at week 26 (69) (Table 2). ADCC activity was not measured in sera at week 6, the only time point when the Env binding magnitude was higher for the gp140-M64U1 group than for the gp140 group. ADCC activity at week 26 significantly correlated with the week 6 Env binding magnitude and off-rate (Fig. 4C and D) but not with the week 26 (contemporary) binding magnitude or off-rate (Table 3). The brisk and avid antibody response may be a biomarker for another underlying (and unmeasured) mechanism that led to enhanced ADCC function, or the early antibody response may have directly affected the immune mechanisms resulting in higher ADCC function. Interestingly, ADCC activity was also found to correlate with the proportions of Env-specific B cells in peripheral blood (69). Week 38 serum neutralizing activity, on the other hand, correlated with the contemporary IgG binding magnitude (Fig. 4A) but not the contemporary IgG Env avidity; it also correlated with a higher off-rate (indicating lower avidity) at the earlier time point of week 6 (Fig. 4B). The discordant correlations of ADCC and neutralizing activities with binding and avidity are in agreement with the observations of Guan et al. (60), suggesting that different Env specificities are involved in ADCC and neutralizing antiviral functions.

Env-specific antibodies were detected in nasal samples from both the gp140 and gp140-M64U1 groups, with no difference in IgG levels between groups. The level of Env-specific IgG in the nasal compartment did not correlate with the level of serum IgG. This may be explained by selective transportation of serum IgG into mucosal compartments, variation in transportation efficiency among animals, or local production of IgG at the mucosal compartments. The Env gp140 protein used in this vaccine study does not contain V2. This was based on an earlier finding of higher titers of cross-reactive neutralizing antibodies in rhesus macaques immunized with SF162 V2-loop-deleted gp140 than in those immunized with SF162 gp140 (33, 61). In light of the RV144 immune correlation found between plasma anti-V2 IgG and a decreased risk of infection (20), further studies to improve upon this vaccine platform could include the addition of the V2 region in the vaccine immunogen to enable induction of V2-specific responses.

In summary, the data from this study indicate that immunization with an Env protein cross-linked to a CD4-mimetic miniprotein (M64U1) induced an accelerated Env binding magnitude and avidity (as early as 2 weeks after the 2nd immunization). In addition, cross-linking of gp140 with M64U1 modulated particular epitope specificities of antibody responses, such as inducing higher C1_104.AE responses in the gp140 group, likely due to alterations in the envelope structure that modulate exposure of this region upon CD4 binding. Lastly, ADCC activities at peak immunity time points (which were larger for the gp140-M64U1 group than for the gp140 group) correlated with the magnitudes and avidities of Env binding responses at an earlier time point, before the ADCC and binding antibody responses reached peak levels. Taken together, these data indicate that structural modification of HIV-1 Env immunogens by mimicking the CD4-bound state can modulate epitope exposure in a way that substantially affects the specificity and function of the elicited antibody responses.

## MATERIALS AND METHODS

### Animal study design.

Rhesus macaques of Chinese origin were housed at the Biomedical Primate Research Center (BPRC), The Netherlands. The study protocol and experimental procedures were approved by the institute's animal ethical care and use committee and were performed in accordance with Dutch law and international ethical and scientific standards and guidelines (69). The study consisted of four groups of 6 animals each (Table 1). One group (gp140 group) received intramuscular immunizations with 100 mg gp140 protein with variable loop 2 (V2) deleted (SF162ΔV2 gp140), administered in adjuvant MF59; the second group (gp140-M64U1 group) received immunizations with 100 mg gp140 cross-linked with the CD4-mimetic protein M64U1 (gp140-M64U1; produced by incubating gp140 with M64U1-SH, which contains an additional sulfhydryl group on the side chain of Lys4, at a gp140/M64U1-SH ratio of 1:3 [38, 42]), also in MF59. In addition, two control groups received either M64U1/MF59-only (50 mg) or mock immunizations. All protein immunizations were delivered intramuscularly at weeks 0, 4, 24, 36, and 107 of study.

### BAMA.

Env-specific IgG and IgA responses in serum and in mucosal samples were measured as previously described (29, 52, 62). For quantification of IgA responses, IgG was depleted from sera by use of HP MultiTrap protein G filter plates (GE Healthcare Life Sciences). Mucosal specimens were filtered, buffer exchanged, and concentrated to equal volumes before measurements of total and specific antibodies. Rectal wash samples were examined, and none had visual blood contamination. The total IgG concentration in each mucosal sample was determined by a macaque total IgG enzyme-linked immunosorbent assay (ELISA), and specific activity was calculated as follows: specific activity = (MFI × dilution)/total antibody. For characterization of CD4 binding site (CD4bs) and CD4-inducible (CD4i) antibodies, a CD4bs and CD4i differential binding antigen panel was used for BAMA and included the wild-type (WT) YU2 gp120 core, resurfaced stabilized core 3 (RSC3), the HXB2 8b core, and mutants of these proteins containing mutations of amino acids that are known to be required for binding by CD4bs or CD4i antibodies (proteins were kindly provided by J. Mascola, Vaccine Research Center). Relative levels of CD4bs and CD4i antibodies were calculated as the WT MFI/mutant MFI ratios for samples that bound to both the WT and mutant with MFI of >100 and at least 3-fold over the MFI of matched baseline (week 0) samples.

### Linear epitope mapping peptide microarray.

Linear epitope mapping was performed as previously described (63, 64), with modifications. Briefly, array slides were provided by JPT Peptide Technologies GmbH (Berlin, Germany) and were made by printing a peptide library designed by B. Korber (Los Alamos National Laboratory) onto epoxy glass slides (PolyAn GmbH, Germany). The library contains overlapping peptides (15-mers overlapping by 12 residues) covering 7 full-length HIV-1 gp160 Env consensus sequences (clades A, B, C, and D, group M, CRF1, and CRF2) (64). The sequences of the peptides contained in the peptide library were published previously (65). Three identical subarrays, each containing the full peptide library, were printed on each slide. All serum samples were diluted 1:250 and hybridized to the slides by use of a Tecan HS4000 hybridization workstation, followed by incubation with DyLight 649-conjugated goat anti-rabbit IgG (Jackson ImmunoResearch, PA). Fluorescence intensity was measured using a GenePix 4300 scanner (Molecular Devices) and analyzed with GenePix software. The binding intensity of the postimmunization serum for each peptide was corrected with its own background value, which was defined as the median signal intensity of the prebleed serum for that peptide plus 3 times the standard error among the 3 subarrays on the slide.

### SPR test for binding avidity.

Surface plasmon resonance (SPR) tests were performed as previously described (20, 66), using BIAcore 4000 instruments. The binding dissociation rate constant (*K_d_*) and binding magnitude (in response units [RU]) were measured for IgGs purified from NHP sera, at 200 μg/ml, against a panel of HIV-1 Env glycoproteins, including ConS gp140, SF162 gp140ΔV2, MN gp120, and gp41 MN. Env proteins were immobilized as previously described, and the avidity score was calculated by determining the RU/*K_d_* value (20).

### Neutralization assays.

Virus neutralization assays were performed on the TZM-bl cell line, using replication-competent or pseudotyped viruses grown in human PBMC as previously described (67). Serial dilutions of serum samples were tested for neutralization of a panel of tier 1 (SHIV-SF162P4 and SHIV-1157iEL-p [replication-competent viruses]) and tier 2 (SHIV-SF162P3.5 and SHIV-89.6P.18 [pseudotyped viruses] as well as SHIV-89.6 and SHIV-1157ipd3N4 [replication-competent viruses]) SHIVs in TZM-bl cells.

### ADCC assays.

ADCC assays were performed as previously described by Pollara et al. (59), using CEM.NKR_CCR5_ cells coated with recombinant HIV-1 SF162 gp120 as target cells and PBMC obtained from an HIV-seronegative donor as effector cells. The ADCC-mediating antibody titer was defined as the reciprocal of the highest dilution indicating a positive granzyme B (GzB) response (>8% GzB activity) after background subtraction, as previously described (59).

### Statistical analysis.

Differences in the levels of antibody responses between the 2 vaccine groups or between vaccine and control groups were tested using the Wilcoxon rank sum exact test, with the false discovery rate (FDR) controlled using the Benjamini-Hochberg method (68), performed with SAS. Correlations between binding antibody responses (binding magnitudes from BAMA and epitope mapping assays and dissociation rates from SPR assays) and antibody functions (ADCC and neutralization assays) were tested using the Spearman correlation test, with FDR controlled using the Benjamini-Hochberg method.
